# Lignin provides mechanical support to herbaceous peony (*Paeonia lactiflora* Pall.) stems

**DOI:** 10.1038/s41438-020-00451-5

**Published:** 2020-12-28

**Authors:** Daqiu Zhao, Yuting Luan, Xing Xia, Wenbo Shi, Yuhan Tang, Jun Tao

**Affiliations:** 1 College of Horticulture and Plant Protection, Yangzhou University, Yangzhou, 225009 P.R. China; 2 Joint International Research Laboratory of Agriculture and Agri-Product Safety, The Ministry of Education of China, Yangzhou University, Yangzhou, 225009 P.R. China

**Keywords:** Plant physiology, Plant development

## Abstract

Stem bending caused by mechanical failure is a major constraint for high-quality herbaceous peony (*Paeonia lactiflora* Pall.) cut flowers, but little is known about the underlying factors. In this study, two *P. lactiflora* cultivars, Xixia Yingxue (bending) and Hong Feng (upright), were used to investigate differences in stem bending. The results showed that the stem mechanical strength of Hong Feng was significantly higher than that of Xixia Yingxue, and the thickening of the secondary cell wall and the number of thickened secondary cell wall layers in Hong Feng were significantly higher than those in Xixia Yingxue. Moreover, compared with Xixia Yingxue, Hong Feng showed greater lignification of the cell wall and lignin deposition in the cell walls of the sclerenchyma, vascular bundle sheath and duct. All three types of lignin monomers were detected. The S-lignin, G-lignin, and total lignin contents and the activities of several lignin biosynthesis-related enzymes were higher in Hong Feng than in the other cultivar, and the S-lignin content was closely correlated with stem mechanical strength. In addition, 113,974 full-length isoforms with an average read length of 2106 bp were obtained from the full-length transcriptome of *P. lactiflora* stems, and differential expression analysis was performed based on the comparative transcriptomes of these two cultivars. Ten lignin biosynthesis-related genes, including 26 members that were closely associated with lignin content, were identified, and multiple upregulated and downregulated transcription factors were found to positively or negatively regulate lignin biosynthesis. Consequently, lignin was shown to provide mechanical support to *P. lactiflora* stems, providing useful information for understanding the formation of *P. lactiflora* stem strength.

## Introduction

Stem bending or lodging caused by stem mechanical failure is one of the most important agronomic problems and can cause substantial economic losses associated with the quality and yield of agricultural products. For example, bending of *Gerbera jamesonii* cut flowers reduces their ornamental value^1^, lodging contributes to poor grain yield and quality of wheat^2^, and mechanical bending induces tension wood formation in *Liriodendron tulipifera*^3^. Stem mechanical strength is a complex trait influenced by many factors and is closely related to the morphological, structural, and physiological characteristics of stems; increasing stem mechanical strength can improve resistance to stem bending and lodging^4,5^. Previous studies have shown that morphological characteristics, including stem diameter, stem weight, and internode length, can affect the stem mechanical strength of rye^6^, switchgrass, and miscanthus^7^. Moreover, the anatomy of the stem, which primarily depends on the morphology and distribution of mechanical and transduction tissues and the degree of the development of the xylem, can contribute to stem mechanical strength^4^. In addition, the stem chemical constituents, including cellulose, hemicellulose, and lignin, have an important role in the stem mechanical strength^8^.

Lignin is an integral structural component that is deposited together with cellulose and hemicellulose in the secondary cell walls in vascular plants^9^. Previous studies of sudangrass^10^, chrysanthemum^11^, kodo millet^12^, and rice^13^ have shown that reductions in the lignin content caused the stem mechanical strength to decrease, suggesting that lignin is closely tied to stem mechanical strength. Moreover, lignin is composed of guaiacyl, syringyl, and p-hydroxyphenyl units derived from monolignol precursors (*p*-coumaryl alcohol [the H unit], coniferyl alcohol [the G unit], and sinapyl alcohol [the S unit], respectively)^14^. Previous studies showed that the increase in wheat planting density resulted in substantial reductions in S-lignin but no changes in G-lignin, which caused a decrease in the mechanical strength^15^. In studies involving the flexible culm1 mutant of rice and the mechanical bending *Liriodendron tulipifera*, S-lignin was shown to decrease significantly^3,13^. In addition, enzymes, such as phenylalanine ammonia-lyase (PAL), tyrosine ammonia-lyase (TAL), cinnamate 4-hydroxylase (C4H), coumarate 3′-hydroxylase (C3’H), caffeic acid *O*-methyltransferase (COMT), ferulate 5-hydroxylase (F5H), 4-coumarate: CoA ligase (4CL), caffeoyl-CoA *O*-methyltransferase (CCoAOMT), hydroxycinnamoyl CoA (HCT), cinnamoyl-CoA reductase (CCR), cinnamyl alcohol dehydrogenase (CAD), polyphenol oxidase (PPO), and peroxidase (POD) are involved in catalyzing many steps of the lignin biosynthesis pathway^16^, and significant correlations have been found between the activities of these enzymes and the lignin contents of stems^17–19^.

Herbaceous peony (*Paeonia lactiflora* Pall.) is a popular ornamental plant species often used to arrange flower borders, flower beds, potted plants, etc. In addition, *P. lactiflora* has also emerged as a high-end cut flower in the international market in recent years and requires thick and upright stems. However, many *P. lactiflora* cultivars cannot be used as cut flowers because they have elongated stems and droopy flowers. Therefore, studying how the stem mechanical strength of *P. lactiflora* forms is important. To date, some progress has been made in study of *P. lactiflora* stem mechanical strength. For example, Li et al.^20,21^. found that *P. lactiflora* stem mechanical strength was significantly positively correlated with stem diameter, stem weight, and calcium and silicon contents. Subsequently, lignin was found to be the main factor affecting *P. lactiflora* stem mechanical strength^22–24^. Moreover, foliar spraying of calcium and silicon could significantly improve *P. lactiflora* stem mechanical strength by increasing the lignin content^25–27^. However, the relationships between the synthesis, deposition and structure of lignin and the mechanical strength of *P. lactiflora* stem need to be further studied. To explore the physiological, biochemical, and molecular mechanisms underlying the bending resistance of stems and to clarify the influence of lignin on stem mechanical strength, two *P. lactiflora* cultivars with different uprightness were used to investigate the differences in their morphological indices and mechanical strength; their secondary cell wall and its lignification; their lignin synthesis, deposition and structure; and their transcriptome sequences (via RNA-seq). The present study was conducted to provide a theoretical basis for understanding the formation of *P. lactiflora* stem strength.

## Materials and methods

### Plant materials

Two *P. lactiflora* cultivars with different uprightness, Xixia Yingxue (bending) and Hong Feng (upright), were used in this study^20^. They were planted in the field of National Germplasm Repository of Yangzhou University, Jiangsu Province, China (32°39′N, 119°42′E). The top 12 cm of the stems in the flower-bud stage (S1), unfolded-petal stage (S2), and the full-bloom stage (S3) were collected, and their morphological indices were determined. Subsequently, the part between the top 5 cm and top 8 cm of the stems was fixed in 2.5% glutaraldehyde solution and FAA fix solution for microstructure, lignification, and lignin deposition observations, and the other part of the stems was immediately frozen in liquid nitrogen and then stored at −80 °C.

### Morphological indices and mechanical strength determination

Plant height, stem diameter, stem weight, flower diameter and flower weight were measured by a micrometer scale and balance, respectively. Moreover, a universal NK-2 digital force testing device (Zhejiang Hui’er Instrument and Equipment Co., Ltd., China) was used to measure the mechanical strength of the stems.

### Secondary cell wall observations

Secondary cell walls of the stems were observed by environmental scanning electron microscopy (Philips XL-30 ESEM, Holland) and transmission electron microscopy (TEM) (Philips CM100, Holland). The specific details can be found in a study by Zhao et al.^28^.

### Lignification and lignin deposition observations

For paraffin sectioning, 3–4 mm cross-cut stems from the part between the top 5 and 8 cm were selected and then fixed in FAA fix solution for more than 24 h. The procedure of preparing the paraffin sections was as follows: first, the samples were soaked in mixtures of 50% ethanol:glycerin (1:1, v/v) to soften for 12 h. Then, a gradient of ethanol solutions (50, 70, 90 and 100%, 30 min each) was used for sample dehydration, followed by a gradient of mixtures of dimethylbenzene:ethanol (1:3, 1:1, 3:1, v/v, 30 min each). Finally, mixtures of dimethylbenzene:chloroform (9:1, v/v) were used to complete the last dehydration; this repeated twice, and each time lasted for 30 min. Second, each sample was transferred to a 5 mL centrifuge tube, added to a paraffin:dimethylbenzene mixture (1:1, v/v), incubated at 42 °C for 12 h, and then embedded in pure paraffin. Each sample was then incubated at 42 °C for 24 h, after which it was embedded in pure paraffin. Each sample was then incubated at 50 °C for 3 h and then embedded in pure paraffin; it was subsequently incubated at 60 °C for 3 h, and replaced by a new centrifuge tube and covered using pure paraffin; finally, the sample was incubated at 60 °C for 2 h, and replaced using pure paraffin three times. Third, each sample was put in a metal mold, after which a plastic mold was used to cover the sample for fixing. Liquid paraffin was slowly added until it overflowed, after which the metal mold was then put on a cooling table until it solidified; finally, the embedded sample was removed. After trimming the paraffin block to a suitable size, each sample was placed on a microtome and sliced. The cut paraffin section was placed in a 40 °C water bath for expansion, and after it had fully unfolded, it was attached to a glass slide and heated at 45 °C. Finally, the paraffin section was soaked in dimethylbenzene for 10 min, which was repeated once. Afterward, the section was soaked in a mixture of methylbenzene:ethanol (1:1, v/v) for 5 min and a gradient of ethanol solutions (90, 80, 70, and 50%, 5 min each) was used for rehydration. In addition, the prepared paraffin section was used for subsequent staining and observations.

Based on the prepared paraffin sections, the lignification and lignin deposition were observed using the crystal violet staining method and phloroglucinol staining method, the specific details of which are available in the study by Zhao et al.^28^.

### Lignin structure analysis

Lignin structure was analyzed using Fourier transform infrared (FTIR) and two-dimensional heteronuclear single quantum coherence (2D-HSQC). The lignin powder of the stem was extracted by the high boiling solvent method^29^.

For FTIR, the specific details are available in the study by Zhao et al.^28^. For 2D-HSQC, 20 mg of lignin powder was first dissolved in 0.5 mL of DMSO-*d6*. The spectra were then recorded at 25 °C using a nuclear magnetic resonance spectrometer (Bruker AVANCE 600, Switzerland). The spectral widths were 3,497 and 18,750 Hz for the ^1^H and ^13^C dimensions, respectively. The number of sampling points in the ^1^H dimension was 1,024, the relaxation time was 1.5 s. The number of collected sampling points in the ^13^C dimension was 256, and the ^1^*J*_CH_ was 145 Hz. Prior to Fourier transformation, the data matrices were zero-filled to 1024 points in the ^13^C dimension. The central solvent (DMSO) peak was used as an internal chemical shift reference point (*δ*_C_/*δ*_H_ of 39.5/2.49).

### Lignin content measurements

Resolution of the lignin polymer was performed by alkaline nitrobenzene oxidation. A total of 0.05 g of dry lignin powder was placed in a 25 mL polytetrafluoroethylene sealed tank containing 5 mL of NaOH solution (2 mol/L) and 0.5 mL of nitrobenzene and reacted at 170 °C for 3.5 h. After cooling, the reaction mixture was transferred to a 100 mL erlenmeyer flask, and 0.2 mL of ethyl vanillin solution (4 mg/mL) was added as an internal standard. The reaction mixture was extracted three times using 30 mL of a dichloromethane:ethyl acetate mixture (1:1, v:v), and the pH of the resulting aqueous phase was adjusted to 3.0–4.0 with 6 mol/L hydrochloric acids, which was extracted three times again using 30 mL of a dichloromethane:ethyl acetate mixture (1:1, v:v), and a solid residue was obtained after rotary evaporation at 40 °C. The residue was diluted with 10 mL of methanol, filtered through a 0.22-µm organic filter membrane, and then analyzed via high-performance liquid chromatography (HPLC) (Agilent 6460, USA). *p*-Hydroxybenzaldehyde, vanillin, and syringaldehyde were as standards for the determination of H-lignin, G-lignin, and S-lignin, respectively.

### Lignin biosynthesis-related enzyme activity measurements

First, the crude extracts of the stem were extracted according to the methods in our previous study^28^. Afterward, PAL, C4H, CAD, TAL, and PPO activities were measured using reagent kits, respectively (Keming Biotechnology Co., Ltd., China). Their activity units were expressed in units per gram of FW, and U indicated the catalytic activity of the enzyme.

### PacBio cDNA library construction, sequencing, and data analysis

Total RNA was extracted using TRIzol reagent (Invitrogen, CA, USA). One cDNA library was constructed by mixing equal amounts of the stems of Xixia Yingxue and Hong Feng at three different developmental stages. First-strand cDNA was synthesized from total RNA using a SMARTer polymerase chain reaction (PCR) cDNA Synthesis Kit (Clontech, USA), and then the first-strand cDNA was synthesized with SMARTScribe Reverse Transcriptase. Subsequently, a large amount of double-stranded cDNA was produced. The cDNA was then measured using Qubit HS (Life Technologies, USA) and an Agilent 2100 Bioanalyzer (Agilent Technologies, CA). Finally, a single molecular real-time (SMRT) bell library was constructed with a PacBio DNA Template Prep Kit, and sequencing reactions were performed using the PacBio RS II Platform (Shenzhen, China).

Raw sequencing data (raw reads) were initially processed with the SMRTlink analysis package. Circular consensus sequences (CCSs) were generated from the initial data. Raw reads with a 5′-primer, 3′-primer, and poly-A tail were defined as the full-length reads. Transcript clusters were found using Iterative Clustering for Error Correction, and LoRDEC software was used to correct nucleotide mismatches. Any redundancy was then deleted by using CD-HIT software^30^ (parameters: -c 0.98, -T 6, -G 0, -aL 0.90, -AL 100, -aS 0.98, -AS 30) based on the sequence similarity. Finally, the polished, high-quality, full-length isoforms were compared against the content in public protein databases, such as the nonredundant protein (NR), nucleotide sequence (NT), Protein family (Pfam), euKaryotic Orthologous Groups (KOG), SwissProt, Kyoto Encyclopedia of Genes and Genomes (KEGG), and Gene Ontology (GO) databases.

### Illumina cDNA library construction, sequencing, and data analysis

Eighteen cDNA libraries were constructed using the stems of Xixia Yingxue and Hong Feng at three different developmental stages (three replicates). Purified RNA was fragmented into small pieces with a fragment buffer. The fragmented RNA was then used as a template to synthesize the first-strand cDNA in a first-strand reaction system via PCR, and second-strand cDNA was also synthesized. Magnetic beads were used to purify the reaction products, and then an A-tailing mixture and RNA index adapters were added for end-repair following the Illumina cDNA library construction protocol. PCR was used to amplify the cDNA fragments in conjunction with adapters, and the products were purified by AMPure XP Beads (Beckman, USA). An Agilent 2100 Bioanalyzer (Agilent Technologies, Palo Alto, CA) was used to assess the quality and quantity of the cDNA, after which the qualified library was amplified by an Illumina cBot (Illumina, CA) instrument to generate clusters of the flowcell. The amplified flowcell was subsequently paired-end sequenced on the Illumina HiSeq X-Ten Platform (Shenzhen, China).

The expression level of each gene was calculated by using RSEM^31^ and converted into fragments per kilobase per million fragments (FPKM) according to the read counts. Generally, differential expression analysis was performed by using DESeq2^32^, with parameters including a fold change ≥2.0 and a false discovery rate ≤0.001.

### Gene expression analysis

Quantitative real-time PCR (qRT-PCR) was used to analyze gene expression levels with a Bio-Rad CFX Connect^TM^ Optics Module (Bio-Rad, USA), and their values were calculated according to the 2^−ΔΔCt^ comparative threshold cycle (Ct) method^33^. The specific details of qRT-PCR are available in our previous study^28^. All the primers used are listed in Table S1.

### Statistical analysis

All the data are the average values of at least three replicates and their standard deviations. The variance of the results was analyzed with the SAS/STAT statistical analysis package (version 6.12, SAS Institute, Cary, NC, USA).

## Results

### Stem mechanical strength and morphological indices

**Fig. 1:**
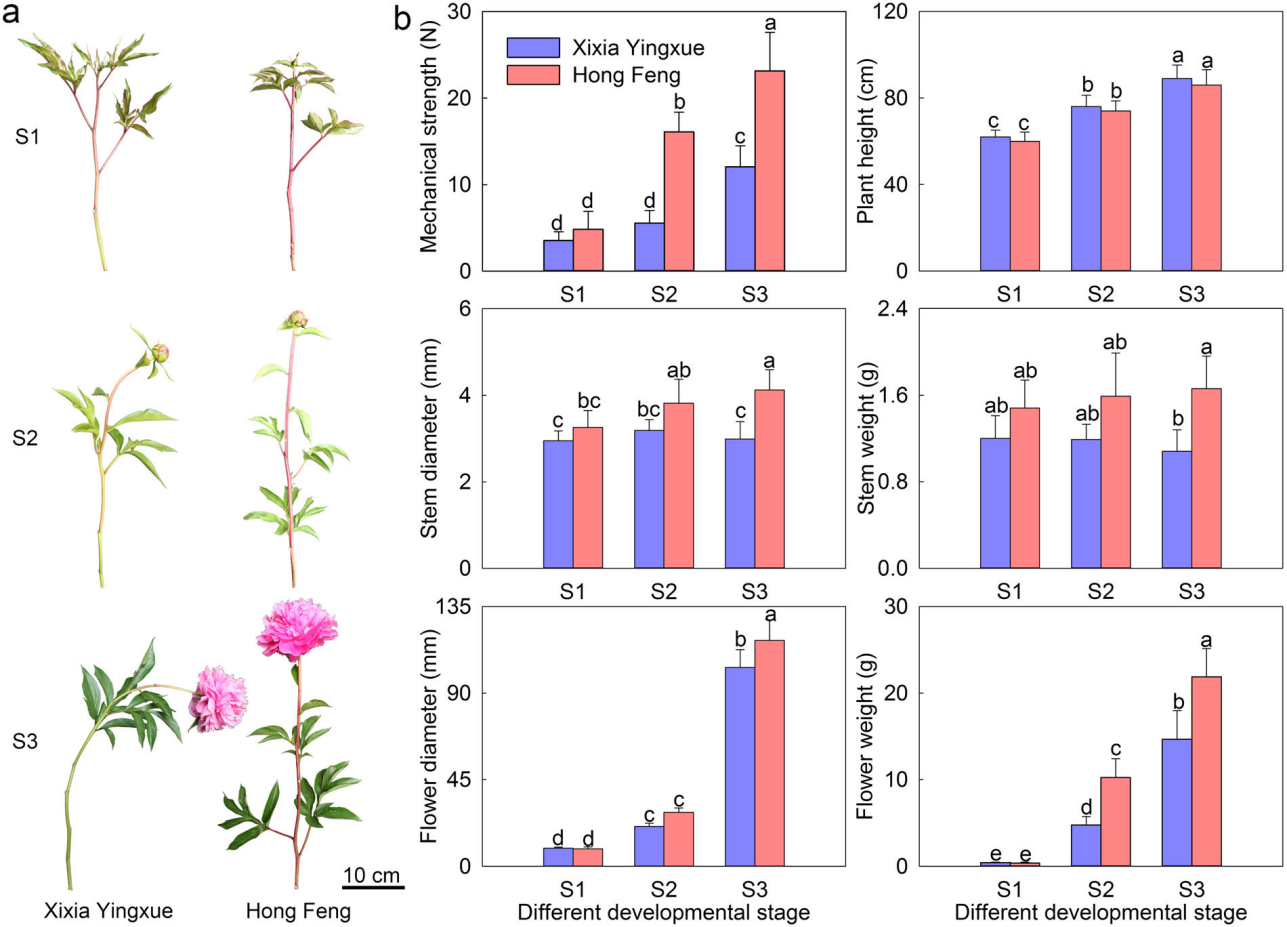
Images and morphological indices of two *P. lactiflora* cultivars at three different developmental stages. **a** Images of two *P. lactiflora* cultivars; **b** Morphological indices. S1, flower-bud stage; S2, unfolded-petal stage; S3, full-bloom stage. The values represent the means ± SDs, and different letters indicate significant differences (*P* < 0.05)

Two *P. lactiflora* cultivars with different uprightness were used in this study (Fig. 1a). The stem of Xixia Yingxue began to bend at S2, while the flower drooped significantly at S3. However, the stem of Hong Feng was straight throughout the developmental process. Their stem mechanical strength and morphological indices were then measured (Fig. 1b). The stem mechanical strength of both cultivars gradually increased as the stem developed, and Hong Feng was significantly higher than Xixia Yingxue except at S1. Moreover, the plant height of these two cultivars was essentially the same, while the stem diameter, stem weight, flower diameter, and flower weight were significantly different at S3.

### Secondary cell wall

**Fig. 2:**
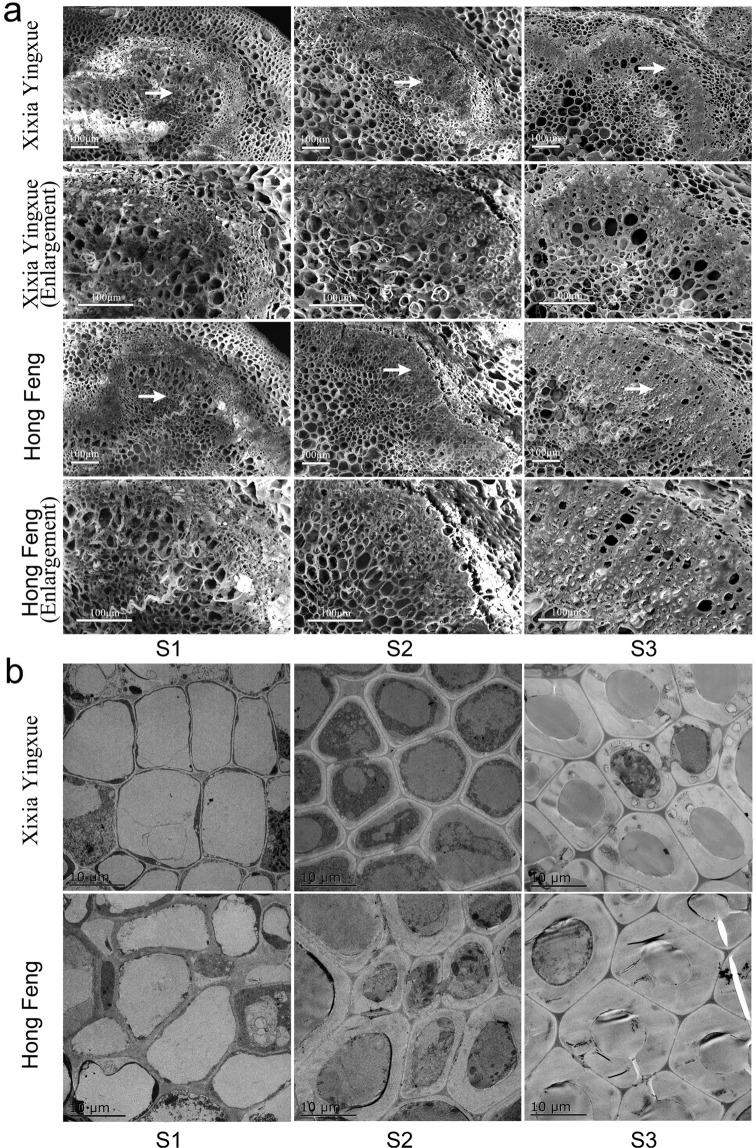
Observations of the secondary cell walls of two *P. lactiflora* cultivar stems at three different developmental stages. **a** The layers of thickened secondary cells were observed via SEM. Micrographs of partially enlarged regions are marked by white arrows. **b** The thickness of secondary cell walls was observed via TEM. S1, flower-bud stage; S2, unfolded-petal stage; S3, full-bloom stage

As shown in Fig. 2a, in addition to the absence of secondary cells in the early stage of stem development (S1), the secondary cell walls of Xixia Yingxue and Hong Feng continued to thicken as the stem developed, the number of thickened cell wall layers increased continuously, and the number of thickened cell wall layers was significantly greater in Hong Feng than in Xixia Yingxue. Moreover, the thickening of the secondary cell walls of the stem was more pronounced under TEM (Fig. 2b). The stems at S1 were primarily composed of parenchyma cells. The secondary cell walls of Xixia Yingxue began to thicken at S2, but the degree of thickening was not yet evident, while the secondary cells of Hong Feng had matured and differentiated at S2. In addition, at S3, the degree of thickening of the secondary cell wall in both cultivars was greater than that observed at S2 and was generally more pronounced in Hong Feng than in the other cultivar.

### Lignification of the cell wall and lignin deposition

**Fig. 3:**
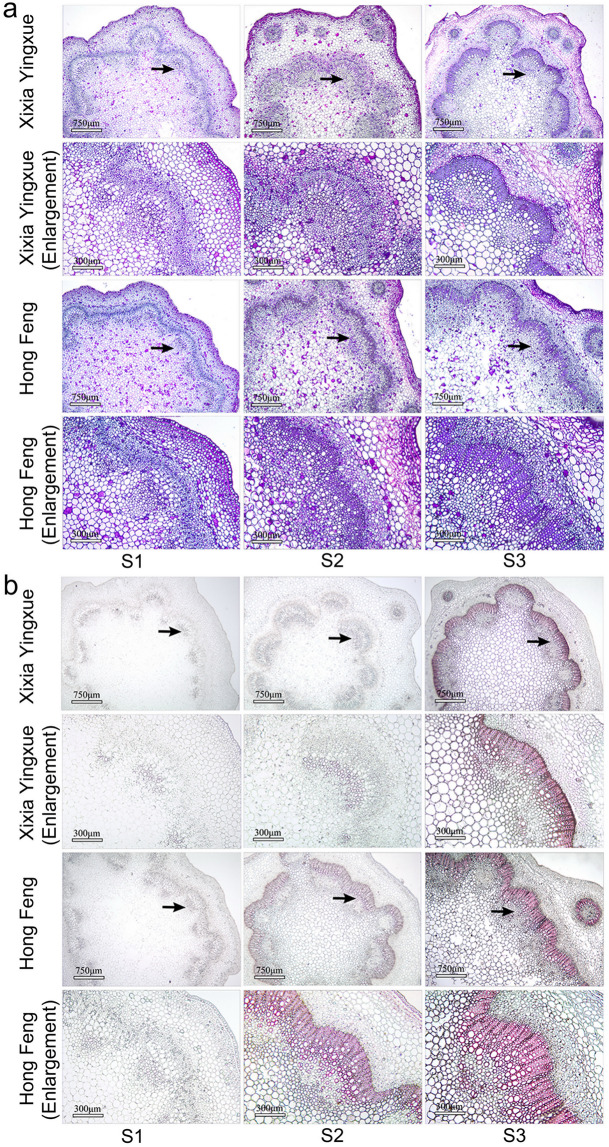
Histochemical staining of the stems of two *P. lactiflora* cultivars at three different developmental stages. **a** Cell wall lignification. **b** Lignin deposition. The micrographs of partial enlargement of regions are marked by black arrows. S1, flower-bud stage; S2, unfolded-petal stage; S3, full-bloom stage

Lignification of the cell wall and lignin deposition were further observed using histochemical staining. First, the stem transverse sections were stained using crystal violet, and lignified cell walls were stained violet (Fig. 3a). The degree of lignification of the cell walls of the Hong Feng and Xixia Yingxue stems increased as the stem developed, and the distribution of lignified cell walls increased. When compared with that of Xixia Yingxue, the degree of lignification of Hong Feng stems was relatively higher during stem development.

Wiesner staining is known to react with cinnamaldehyde residues in lignin, and the color intensity was consistent with the lignin content. At S1, the cell walls of both Xixia Yingxue and Hong Feng stems were similarly light in color, with only a small amount of lignin deposition, and there was little difference between them. At S2 and S3, lignin was mainly deposited in the cell walls of sclerenchyma, vascular bundle sheath, and duct. Compared with Xixia Yingxue, Hong Feng presented a greater stained area and a deeper fuchsia color, indicating that the degree of lignin deposition and the lignin content in Hong Feng were greater than those in Xixia Yingxue (Fig. 3b).

### Lignin structure

**Fig. 4:**
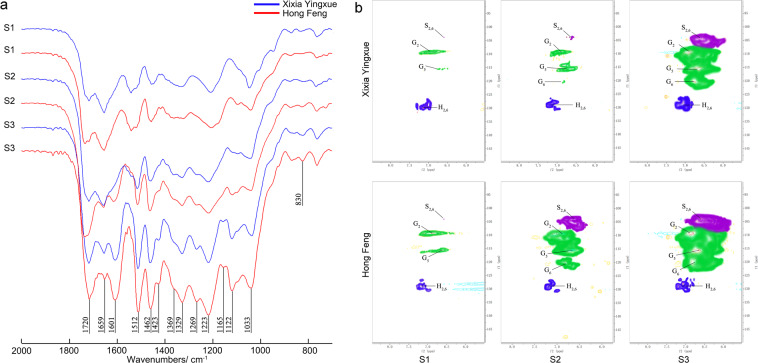
FTIR spectrum and 2D-HSQC of lignin extracted from two *P. lactiflora* cultivar stem at three different developmental stages. **a** FTIR spectrum of lignin; **b** 2D-HSQC results for lignin. S_2,6_ signals represent S-lignin; G_2_, G_5_, and G_6_ signals represent G-lignin; and H_2,6_ signals represent H-lignin. S1, flower-bud stage; S2, unfolded-petal stage; S3, full-bloom stage

FTIR spectrum and 2D-HSQC were used to assess the lignin structure of the stems. The lignin FTIR spectra of Xixia Yingxue and Hong Feng were similar, and the characteristic absorption peaks of the lignin functional groups were primarily concentrated in the range of 1800–900 cm^−1^ (Fig. 4a). Absorption peaks at 1601 cm^−1^ and 1512 cm^−1^ indicated benzene skeleton vibration, absorption peaks at 1329 cm^−1^ and 1125 cm^−1^ indicated syringyl ring (S unit), absorption peaks at 1269 cm^−1^, 1223 cm^−1^, absorption peaks at 1033 cm^−1^ and 834 cm^−1^ indicated guaiacyl ring (G unit), and the absorption peak at 1165 cm^−1^ indicated C=O stretching vibration of *p*-hydroxyphenyl (the H unit). In these two cultivars, H-lignin, S-lignin, G-lignin were detected, and the S-lignin, G-lignin, and total lignin contents increased continuously with stem development.

Moreover, Fig. 4b showed that the main cross signals in the aromatic ring region (*δ*_C_/*δ*_H_ of 90–160/6.0–8.0) corresponded to the aromatic rings of different lignin units, including syringyl, guaiacyl, and *p*-hydroxyphenyl. A C_2,6_–H_2,6_ correlation was observed in the spectra at *δ*_C_/*δ*_H_ 104.1/6.74, representing the S unit. Signals appearing in the spectra at *δ*_C_/*δ*_H_ 111.0/7.01, 114.4/6.73–115.3/6.98, and 119.0/6.82 corresponded to the C_2_–H_2_, C_5_–H_5_, and C_6_–H_6_ correlations, respectively, representing the G unit. Additionally, signals of the C_2_–H_6_ correlation, which represented the H unit, were detected at *δ*127.8/7.22. It could be seen from the obtained signals that H-lignin was at a low level in Xixia Yingxue and Hong Feng, while S-lignin and G-lignin contents increased significantly during the late stages, and these contents in Hong Feng were higher than those in Xixia Yingxue.

### Lignin content and lignin biosynthesis-related enzyme activity

To confirm the above results, the lignin content of the stems was measured using HPLC (Figs. S1, 5a). The total lignin content of Xixia Yingxue and Hong Feng increased with stem development, and Hong Feng had significantly higher lignin content than Xixia Yingxue during the late stages. In terms of the contents of the three lignin monomers, it could be clearly seen that S-lignin and G-lignin were the major lignin components of the stems and constantly increased as the stems developed, and Hong Feng had significantly higher lignin contents than Xixia Yingxue in the late stages. The S/G ratio of these two cultivars increased from S1 to S3, and Hong Feng had a consistently higher S/G ratio compared with that of Xixia Yingxue during the stem development.

**Fig. 5:**
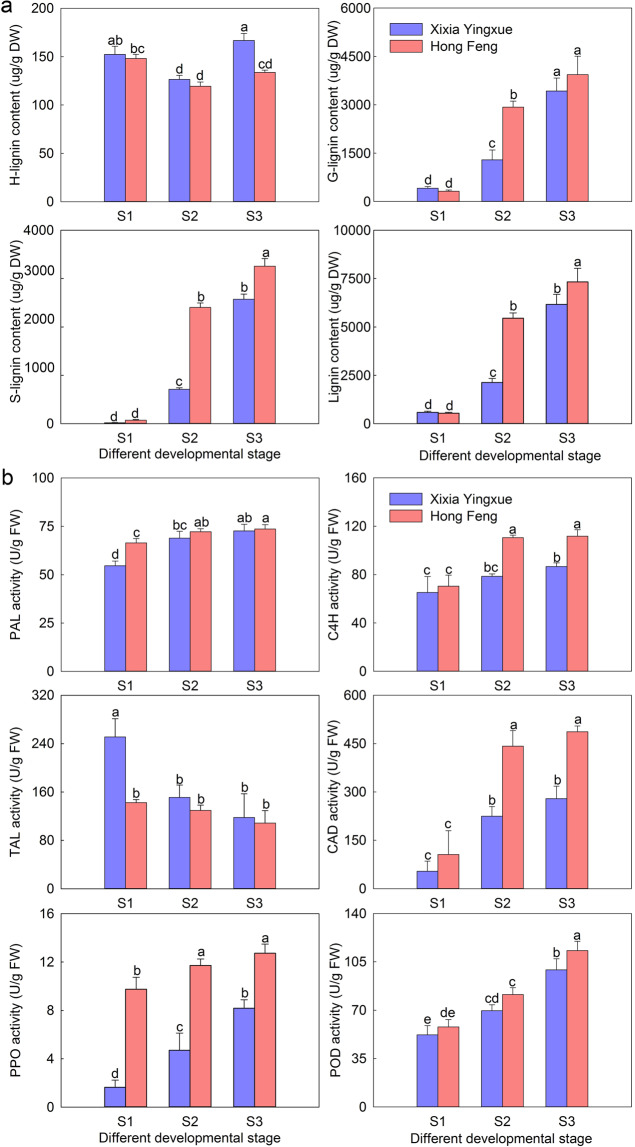
Lignin content and biosynthesis-related enzyme activities in two *P. lactiflora* cultivar stem at three different developmental stages. **a** Lignin content. **b** Lignin biosynthesis-related enzyme activity. S1, flower-bud stage; S2, unfolded-petal stage; S3, full-bloom stage. The values represent the means ± SDs, and different letters indicate significant differences (*P* < 0.05)

The activity of six lignin biosynthesis-related enzymes, PAL, C4H, TAL, CAD, PPO and POD, was then analyzed (Fig. 5b). The PAL, C4H, CAD, PPO and POD activities in Hong Feng were higher than those in Xixia Yingxue, and significant differences between Xixia Yingxue and Hong Feng at S3 were detected for C4H, CAD, PPO and POD activities. However, the TAL activity in these two cultivars decreased, and Hong Feng showed lower TAL activity compared with that of Xixia Yingxue, but the difference was not significant.

### Characterization of the full-length transcriptome and functional annotations

To obtain a better understanding of the molecular mechanisms underlying the lignin regulation of *P. lactiflora* stems, the combined transcript sequencing with SMRT and Illumina sequencing were performed considering no available *P. lactiflora* genome. A mixture of developing stems of Xixia Yingxue and Hong Feng was used to construct a PacBio cDNA library for full-length transcriptome sequencing. A total of 6,859,926 subreads were generated by SMARTlink software, which was classified into 624,682 nonchimeric CCS reads. Of the CCS reads, there were 456,002 full-length nonchimeric reads, with an average length of 1987 bp. Moreover, 191,336 high-quality consensus isoforms (with an average read length of 2108 bp) were corrected using LoRDEC. Finally, any redundancy was removed by CD-HIT software, and 113,974 corrected full-length isoforms (with an average read length of 2106 bp) were ultimately used for subsequent analysis (Table S2).

**Fig. 6:**
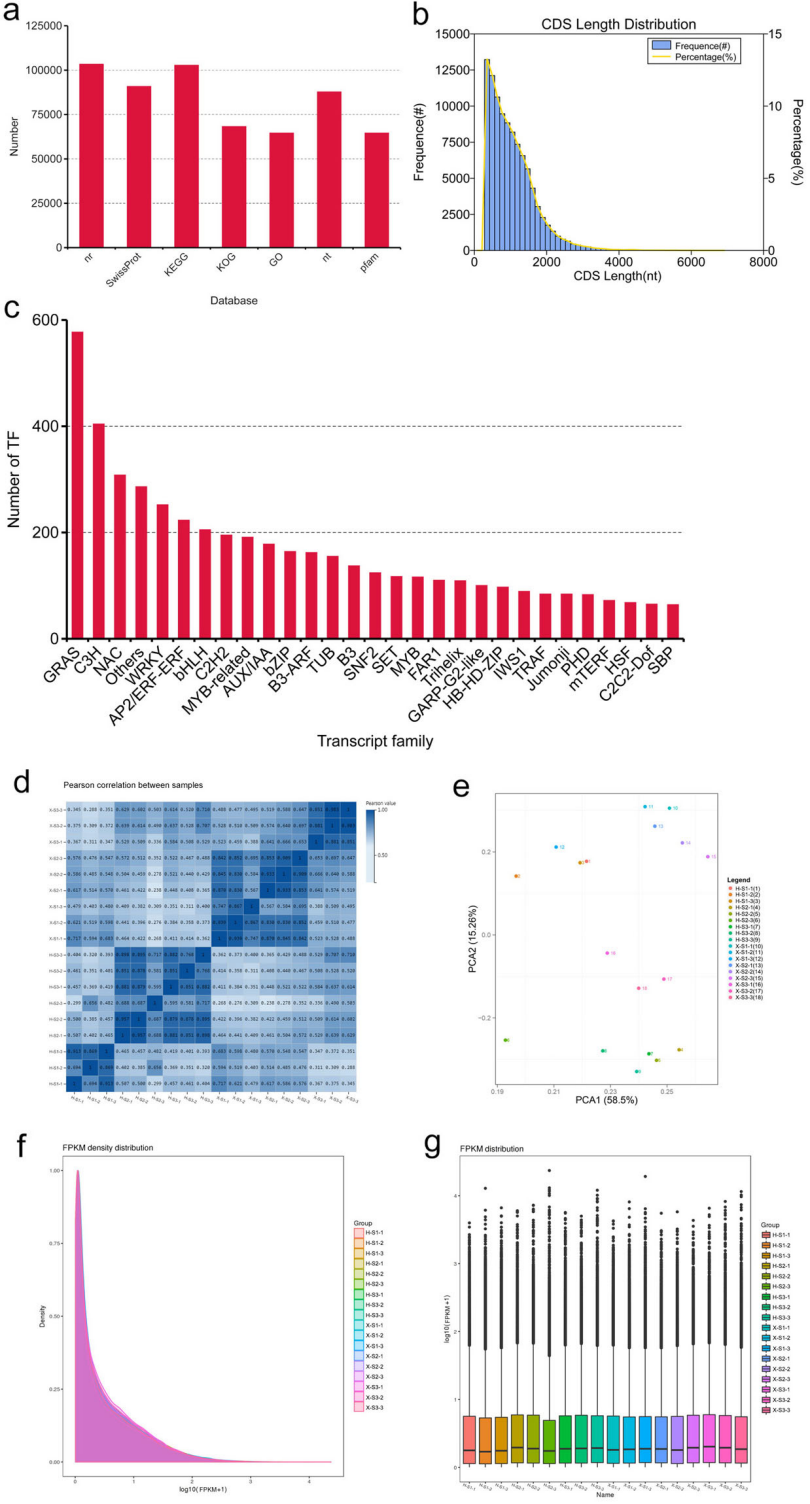
Gene annotation of full-length isoforms and global gene expression analysis. **a** Venn diagram of full-length isoforms annotated in the NR, Swissport, KEGG, KOG, GO, NT, and Pfam public databases. **b** CDS length distribution of full-length isoforms. The *x*-axis represents the CDS length. The *y*-axis represents the number and percentage of the different CDS lengths. **c** TFs prediction of the full-length isoforms. **d** Pearson correlations between eighteen samples based on expression levels. **e** PCA of eighteen samples based on expression levels. **f** Density plot displaying the gene density at different FPKM levels. **g** Box plot of the FPKM distribution among the eighteen samples. X, Xixia Yingxue; H, Hong Feng; S1, flower-bud stage; S2, unfolded-petal stage; S3, full-bloom stage

The full-length isoforms were annotated with seven public reference databases, the NT, NR, GO, KOG, Pfam, KEGG, and SwissProt databases, to obtain comprehensive detailed information on gene function in *P. lactiflora*. First, the majority of full-length isoforms (103,413; 90.73%) had similar sequences in the NR database. The matches to other databases were as follows: 90,856 (79.72%) matched to the SwissProt database, 87,807 (77.04%) matched to the NT database, and 64,611 (56.69%) matched to the Pfam database using BLAST. All the full-length isoforms were subjected to functional annotation and classification. Most could be annotated by the KEGG, KOG, and GO databases. Based on these results, a total of 105,382 full-length isoforms (92.46%) were annotated in at least one database, and 42,601 full-length isoforms (37.38%) were annotated in all databases. Then, we predicted the coding sequence (CDS) and transcription factors (TFs) with coding ability, respectively; the predicted 6009 TFs could be divided into 29 TF families (Fig. 6a–c).

### RNA-seq and differential expression analysis based on the full-length transcriptome

A total of eighteen RNA-seq libraries were constructed for Illumina sequencing. These libraries were generated from the stems of Xixia Yingxue and Hong Feng at three different developmental stages (with three biological replicates). A total of 68.02 M clean reads was obtained on average, which were mapped to the full-length transcriptome using Bowtie2. The average total mapping percentage was 80.25%, and the unique mapping percentage was 9.43% (Table S3). To confirm the reliability of the RNA-seq data, Pearson correlation analysis, principal component analysis, the FPKM density distribution, and a box plot of all the samples suggested high repeatability of the sequencing results (Fig. 6d–g).

Pairwise comparisons were conducted to identify differential expression genes (DEGs) among the samples. It was demonstrated that the number of DEGs was highest in Xixia Yingxue-S2 vs. Hong Feng-S2 (36,336: 18,602 upregulated and 17,734 downregulated DEGs), followed by Xixia Yingxue-S1 vs. Hong Feng-S1 (34,703: 16,627 upregulated and 18,076 downregulated DEGs), and lowest in Xixia Yingxue-S3 vs. Hong Feng-S3 (34,512: 16,072 upregulated and 18,440 downregulated DEGs) (Fig. S2). These DEGs were the most responsive to the mechanical strength of the *P. lactiflora* stems. To validate the accuracy of these DEGs, qRT-PCR was used to analyze the expression levels of nine randomly selected genes, and the results were largely consistent with the RNA-seq data, suggesting that the RNA-seq data were reliable (Fig. S3).

### DEGs related to lignin biosynthesis

**Fig. 7:**
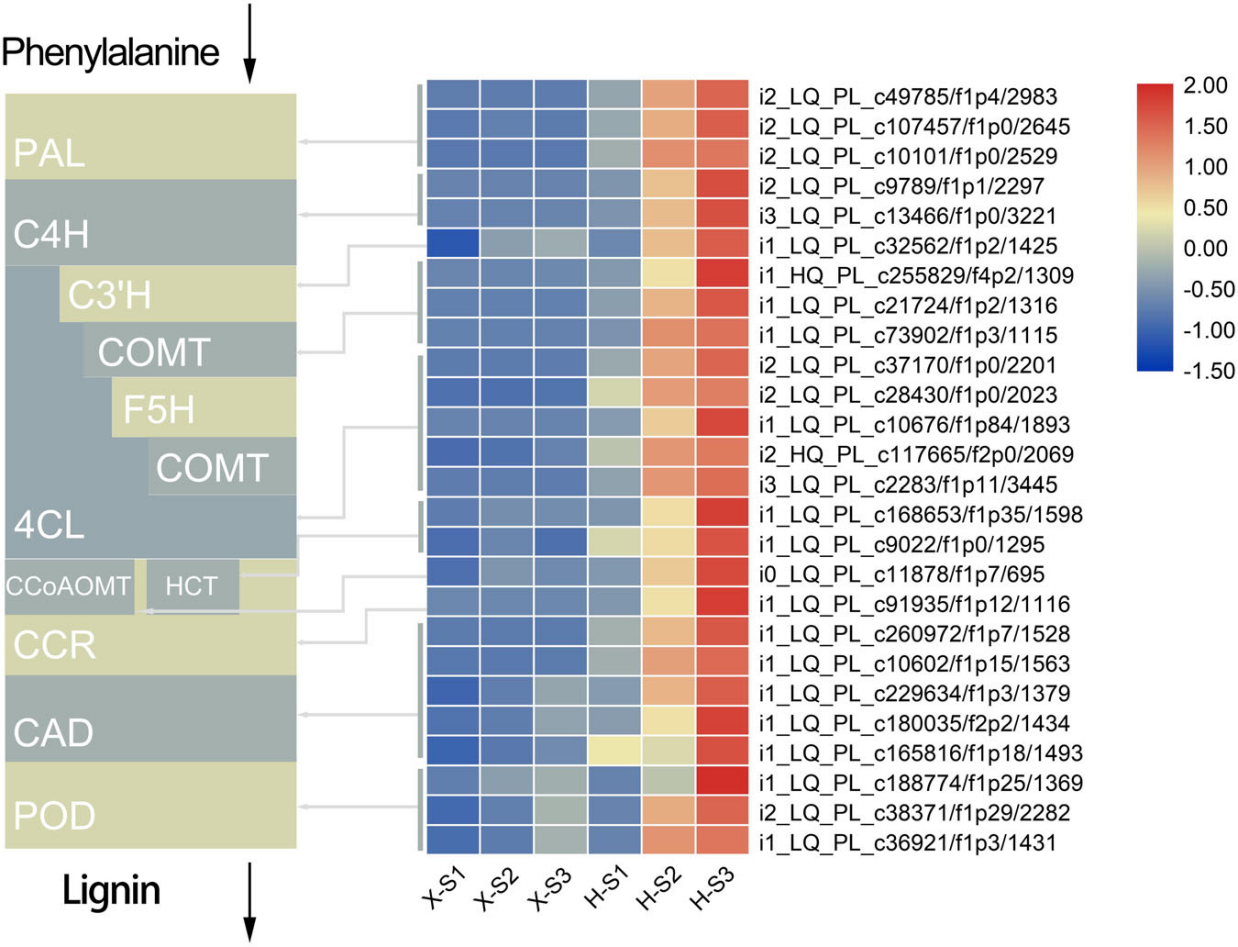
Differential expression of genes involved in the lignin biosynthesis pathway of the stems of two *P. lactiflora* cultivars at three different developmental stages. X, Xixia Yingxue; H, Hong Feng; S1, flower-bud stage; S2, unfolded-petal stage; S3, full-bloom stage

**Fig. 8:**
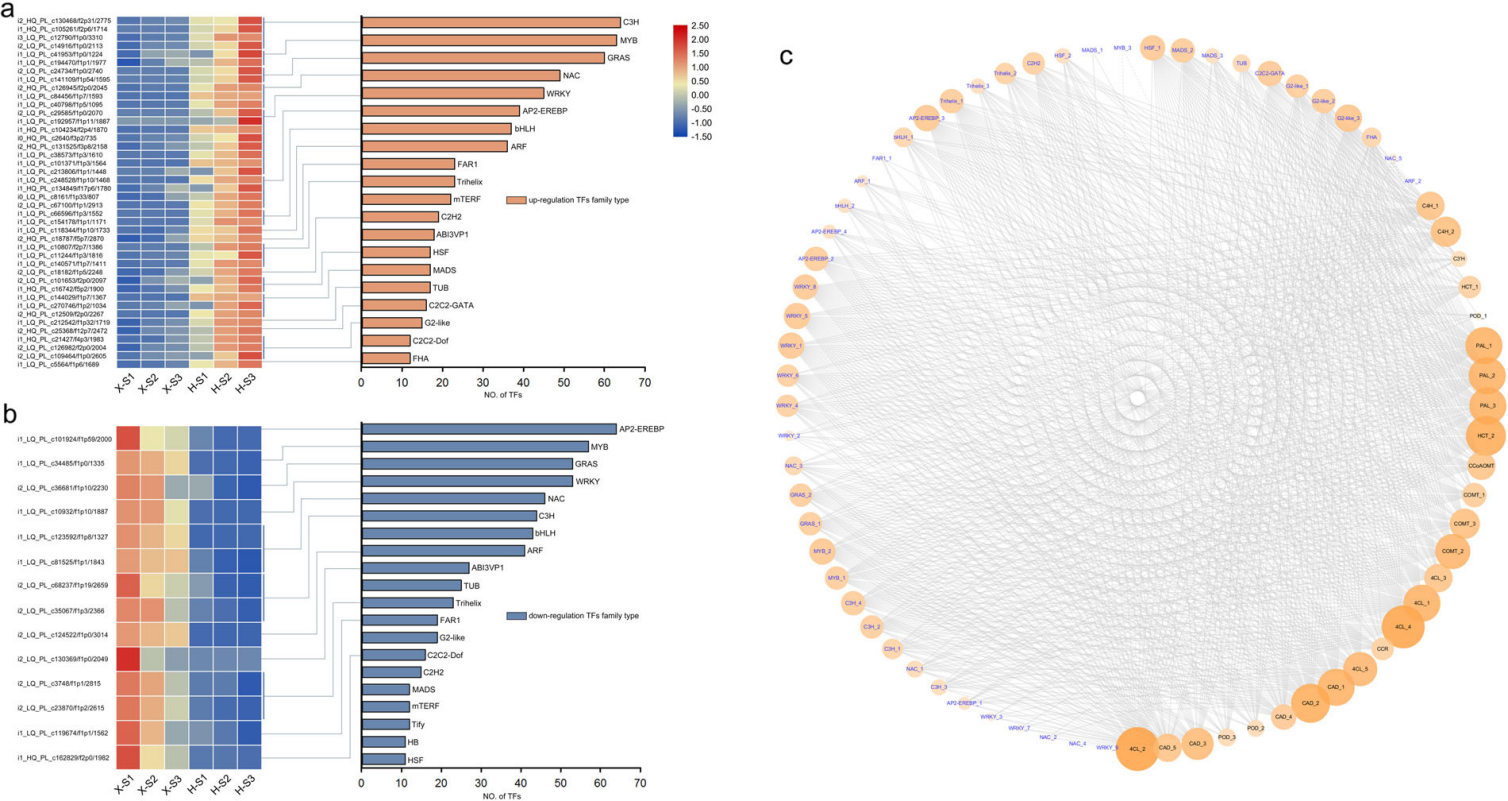
Distribution of differentially expressed TFs of the stems of two P. lactiflora cultivars at three different developmental stages. **a** Upregulated TFs, **b** Downregulated TFs, **c** Co-expression network between candidate structural genes and TFs. X, Xixia Yingxue; H, Hong Feng; S1, flower-bud stage; S2, unfolded-petal stage; S3, full-bloom stage

A fairly large number of markedly regulated DEGs related to lignin biosynthesis were found, including *PAL*, *C4H*, *C3*’*H*, *COMT*, *F5H*, *4CL*, *CCoAOMT*, *HCT*, *CCR*, *CAD*, and *POD*. Subsequently, visual analysis was performed based on their expression levels. The expression levels of these 26 structural genes continuously increased during stem development, and they were higher in Hong Feng than in Xixia Yingxue, which was consistent with change patterns of the lignin content (Fig. 7). In addition, the top 20 TF families with the most DEGs were obtained. Among the TF families whose members presented upregulated expression, the C3H family contained the most DEGs (64), followed by the MYB family (63 DEGs), and the GRAS family (60 DEGs). A total of 42 upregulated TFs distributed in 17 TF families exhibited increasing expression trends in Xixia Yingxue and Hong Feng during stem development, and higher expression levels were observed in Hong Feng, which was consistent with patterns of lignin biosynthesis (Fig. 8a). Among the TF families whose members presented downregulated expression, the AP2-EREBP family contained the most DEGs (64), followed by the MYB family, with 57 DEGs, and the GRAS family, with 53 DEGs. A total of 14 TFs whose expression was downregulated and that were distributed in 11 TF families showed downward expression trends in Xixia Yingxue and Hong Feng during stem development, and higher expression levels were observed in Xixia Yingxue, which was opposite with patterns of lignin biosynthesis (Fig. 8b).

### Co-expression network between lignin-related TFs and structural genes

To identify genes significantly related to stem strength, we constructed a co-expression network of the above differentially expressed TFs and structural genes (Fig. 8c). The correlation of co-efficient between genes was calculated separately using expression data. We set a threshold of 0.9 for a positive correlation and −0.9 for a negative correlation (*p* < 0.05) and then visualized the network using Cytoscape 3.3.0. A total of 47 TFs (42 upregulated ones, 5 downregulated ones) were believed to have a high degree of co-expression relationship with 26 structural genes. The darker the color of the node, the larger radius indicated the stronger connectivity, which meant that the corresponding genes were more important.

Among the structural genes, *4CL_2* (i2_LQ_PL_c28430/f1p0/2023) was linked to the most numbers of TFs, followed by *4CL_4* (i2_HQ_PL_c117665/f2p0/2069), *HCT_2* (i1_LQ_PL_c9022/f1p0/1295) and *CAD_2* (i1_LQ_PL_c10602/f1p15/1563), which were all downstream genes that catalyzed lignin biosynthesis. In the upstream of lignin metabolism, the identified *PAL_1* (i2_LQ_PL_c49785/f1p4/2983), *PAL_2* (i2_LQ_PL_c107457/f1p0/2645), *PAL_3* (i2_LQ_PL_c10101/f1p0/2529), *C4H_1* (i2_LQ_PL_c9789/f1p1/2297), and *C4H_2* (i3_LQ_PL_c13466/f1p0/3221) genes had high connectivity with TFs. The connected TFs included 39 TFs belonging to 16 TFs families, and the WRKY family TFs shared the most connections with the 5 structural genes. Other identified important TFs included 3 NAC family TFs, 2 MYB family TFs, and 4 C3H family TFs, which were thought co-expressed with upstream structural genes (Table S4). At the same time, *NAC_5* (i1_LQ_PL_c81525/f1p1/1843) was identified as the only correlated TF, which was linked to *PAL_3*, showing the opposite expression trend with it.

## Discussion

*P. lactiflora* stem mechanical strength is closely related to related indices of stems and flowers, and significantly positive correlations between stem diameter, flower diameter, and flower weight and stem mechanical strength have been found^22^. In this study, the stem mechanical strength, stem diameter, flower diameter, and flower weight of Xixia Yingxue and Hong Feng increased with plant development, and the stem mechanical strength and stem diameter of Hong Feng were always higher than those of Xixia Yingxue, which was in agreement with the results of a previous study^28^, suggesting that stem diameter could be used as a visual indicator of stem mechanical strength in *P. lactiflora*. Moreover, compared with those of Xixia Yingxue, the flower diameter and flower weight of Hong Feng were higher at S2 and S3, and these results were largely consistent with the trends of the stem mechanical strength, which might be because the rapid development of flowers during the middle and late stages required higher stem mechanical strength to provide support. A previous study in rice showed that the gradual accumulation and output of nutrients occurred simultaneously during the process of stem formation and senescence; specifically, when the accumulation and output were the same and equilibrium had been reached, the nutrients of the stem were sufficient, and the stem mechanical strength was maximized^34^. The nutrients in *P. lactiflora* stems had accumulated to a level that was at or greater than the output level from S1 to S3, the stem diameter increased, and the stem mechanical strength improved continuously, thus providing mechanical support for the developing flowers.

Stem mechanical strength is closely related to stem anatomical structure. In barley, stems with thicker mechanical structures had greater numbers of cell layers and stronger lodging resistance^35^. The stems of lodging-resistant rice cultivars had many structural advantages, such as a thickened stem wall, well-developed mechanical tissue, and a large number of vascular bundles^36^. In *P. lactiflora*, the number of vascular bundles in the stems of Hongyan Zhenghui increased, the cell walls of thick-walled tissue thickened, and the proportion of vascular bundles and pith increased with plant development^22^. In this study, the secondary cell walls of the Xixia Yingxue and Hong Feng stem continued to thicken with the development of the plants, and the number of layers with thickened secondary cell walls also increased; moreover, the number of layers was significantly higher in Hong Feng than in Xixia Yingxue. When *P. lactiflora* was treated with calcium, the stems with increased mechanical strength had significantly thickened secondary cell walls and increased cell layers, indicating that secondary cells played an important role in the formation of stem mechanical strength^25^. In addition, differences in the lignification of the cell walls also have a great impact on stem mechanical strength^35^. In this study, there was a difference in the lignification of cell walls in *P. lactiflora* stems, and the lignification of Hong Feng was higher than that of Xixia Yingxue during stem development. Further observations of the distribution of lignin showed that the lignin coloring intensity gradually increased with stem development, and the coloring range of Hong Feng was greater than that of Xixia Yingxue, which was consistent with the lignin content measurements. In addition, lignin content is closely related to the activity of related enzymes in the lignin biosynthesis pathway. In common buckwheat, the lignin content is significantly positively correlated with PAL, 4CL, CAD and POD activities^37,38^. In the present study, the activities of PAL, C4H, CAD, PPO and POD were higher in Hong Feng than in Xixia Yingxue during stem development, and the activity of all tended to increase, which was in agreement with the obtained lignin content. The trend in TAL activity was the exact opposite of the trend in lignin content in Hong Feng and Xixia Yingxue, indicating that TAL was not important to the lignin biosynthesis of *P. lactiflora* stem.

There are three types of lignin—H-lignin, G-lignin, and S-lignin, and the types and contents of lignin monomers in different plant species are not consistent. G-lignin and S-lignin could be detected in pear fruits^39^, and the lignin from macaúba stems was enriched in S-lignin^40^. In this study, FTIR analysis showed that the characteristic peaks of syringyl, guaiacyl, and *p*-hydroxyphenyl structural units were clearly present, and 2D-HSQC analysis also detected H-lignin, G-lignin, and S-lignin, which indicated that the lignin of the stems comprised G-lignin, S-lignin and H-lignin in *P. lactiflora*. Quantitative analysis of the lignin revealed that G-lignin and S-lignin were the main lignin monomers of *P. lactiflora* stems, and their contents were generally higher in Hong Feng than in Xixia Yingxue. The S/G ratio of these two cultivars increased from S1 to S3, and the S/G ratio of Hong Feng was always higher than that of Xixia Yingxue. Similarly, Pramod et al. observed decreases in the contents of S-lignin and G-lignin in subabul tension wood, and the S/G ratio was significantly lower than that of its opposite wood, which was in agreement with the results of this study^41^. These results indicated that S-lignin played an important role in the formation of *P. lactiflora* stem mechanical strength, which was in agreement with previous studies showing that S-lignin mainly played a role in mechanical support^15,42^.

Because of its large genome and high heterozygosity, genome sequencing has not yet been completed for *P. lactiflora*, which has made the study of this species increasingly difficult. In recent years, third-generation full-length transcriptome sequencing based on PacBio SMRT sequencing technology has facilitated molecular biology research for plants lacking genomic information, and this technique has been used in *Coptis deltoidea*^43^, *Crocus sativus*^44^, and *Olea europaea*^45^. In this study, a mixture of stems of Xixia Yingxue and Hong Feng at different developmental stages was used to construct a PacBio cDNA library for full-length transcriptome sequencing, and 113,974 full-length isoforms with an average read length of 2106 bp were obtained, laying a foundation for identifying excellent genes that regulated the mechanical strength of *P. lactiflora* stems. On this basis, RNA-seq was then used to sequence stems of Xixia Yingxue and Hong Feng at three different developmental stages, and a total of 68.02 M clean reads were obtained on average, with an average total mapping percentage was 80.25%. In addition, a large number of DEGs between these two cultivars were obtained, and these results were confirmed by qRT-PCR.

Genes in the lignin biosynthetic pathway can be divided into three categories. One category contains *PAL*, *C4H*, and *4CL* in the phenylpropanoic acid pathway, and their expression levels are significantly correlated with lignin content^46^. In transgenic plants, low expression levels of these three genes resulted in decreases in lignin content^47,48^. The second category is the lignin monomer biosynthesis-related genes, including *HCT*, *C3*′*H*, *CCoAOMT*, *COMT*, and *F5H*, which influence lignin monomer biosynthesis, determine the proportion of various monomers in lignin, and thus affect the total lignin content to some extent^49^. In jute or *Arabidopsis*, the repression of *COMT* or *F5H* expression resulted in significant decreases in S-lignin^50,51^. Moreover, downstream of the lignin biosynthetic pathway, some genes involved in lignin monomer biosynthesis and polymerization, such as *CCR*, *CAD*, and *POD*^49^, were significantly positively correlated with lignin content^52^. In the present study, 26 DEGs were identified as being related to lignin biosynthesis, including 3 *PAL*, 2 *C4H*, 1 *C3*′*H*, 3 *COMT*, 5 *4CL*, 1 *CCoAMOT*, 2 *HCT*, 1 *CCR*, 5 *CAD*, and 3 *POD* genes. The expression levels of these DEGs related to lignin biosynthesis tended to increase in the stems of Xixia Yingxue and Hong Feng at different developmental stages, and the expression levels of these genes were always higher in Hong Feng than in Xixia Yingxue, which was consistent with the observed changes in lignin content. These genes could provide a foundation for improving stem mechanical strength via genetic engineering. In addition, studies in model plant species have shown that lignin biosynthesis is regulated by multiple TFs, such as the MYB and NAC families^53^. In this study, the expression levels of 42 upregulated TFs distributed in 17 TF families were consistent with the patterns of lignin biosynthesis, while the expression levels of 14 downregulated TFs distributed in 11 TF families were opposite with patterns of lignin biosynthesis.

To further clarify the relationships between TFs and structural genes, we constructed a co-expression network between different genes based on the RNA-seq data. In this network, we identified multiple downstream genes that catalyzed lignin biosynthesis, including *4CL_2*, *4CL_4*, *HCT_2*, and *CAD_2*. We focused on the differentially expressed structural genes upstream of lignin metabolism, and identified a total of 5 structural genes (*PAL_1*, *PAL_2*, *PAL_3*, *C4H_1*, and *C4H_2*) that were highly co-expressed with a large number of TFs. These genes formed 164 co-expression relationships with 39 TFs from 16 TF families. At the transcriptional level, multiple TF families regulate lignin biosynthesis and form complex transcriptional regulatory networks^53^. The NAC family acts as the first-level master switches to control the expression of downstream TFs^54,55^. The second-level of regulators includes many MYB TFs and zinc-finger TFs^56^. In this study, a total of three NAC family TFs, two MYB family TFs, and four C3H family TFs were identified. The final results showed that this TFs might regulate stem lignification via these five structural genes. Among them, *NAC_5* was the only downregulated TF, which was connected to the upstream *PAL_*3. The predicted relationships between TFs and structural genes provided us with very useful information to help us analyze the underlying factors of the stem strength diversity among different cultivars in more depth. Overall, this differentially expressed TFs could be further analyzed via biochemical experiments to characterize their gene functions underlying lignin biosynthesis in *P. lactiflora* stems.

## Conclusions

*P. lactiflora* stems accumulate high amounts of lignin during their development, and this accumulated lignin provides mechanical support to the stems. The thickening of the secondary cell wall, number of thickened secondary cell wall layers, lignin deposition, S-lignin content, and lignin biosynthesis-related enzyme activities were all positively correlated with stem mechanical strength. By comparing the transcriptome profiles of two *P. lactiflora* cultivar stems at different stages, key genes involved in the lignin biosynthetic pathway were identified, and these genes contributed directly to the regulation of lignification in *P. lactiflora* stems. Moreover, the expression profiles of a number of differentially expressed TFs, including three NAC family TFs, two MYB family TFs, and four C3H family TFs were highly correlated with the expression profiles of key lignin biosynthesis-related genes. The findings of this work provide useful information for understanding the formation of *P. lactiflora* stem strength.

## Supplementary information

supplemental materials
