# Supplementary material for: Lignin provides mechanical support to herbaceous peony (Paeonia lactiflora Pall.) stems
Source: Hortic Res. 2020 Dec 28;7:213. doi: 10.1038/s41438-020-00451-5 (PMC7769982; doi:10.1038/s41438-020-00451-5)
Supplement: Supplementary file 1 — supplemental materials [file 41438_2020_451_MOESM1_ESM.doc]

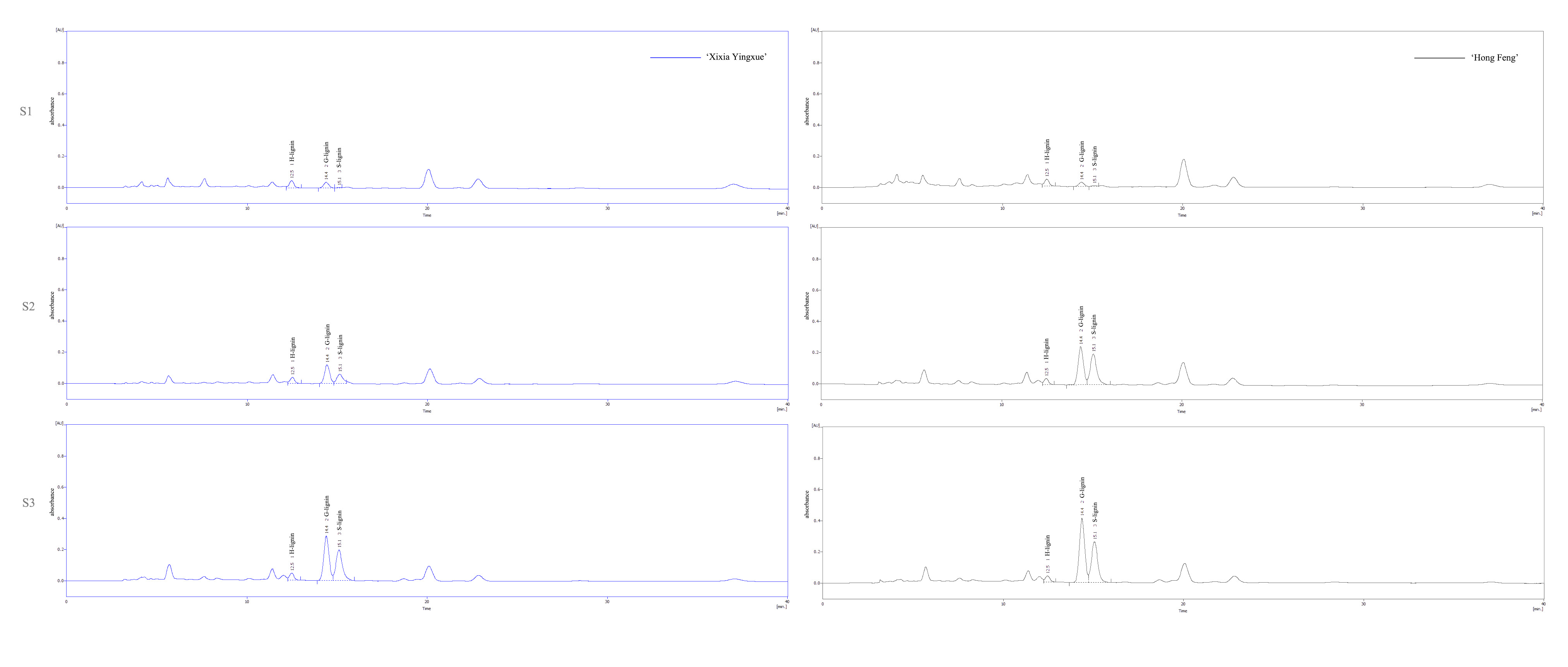


**Fig. S1.** HPLC chromatogram of two *P. lactiflora* cultivars at three developmental stages. S1, flower-bud stage; S2, unfold-petal stage; S3, full-bloom stage.


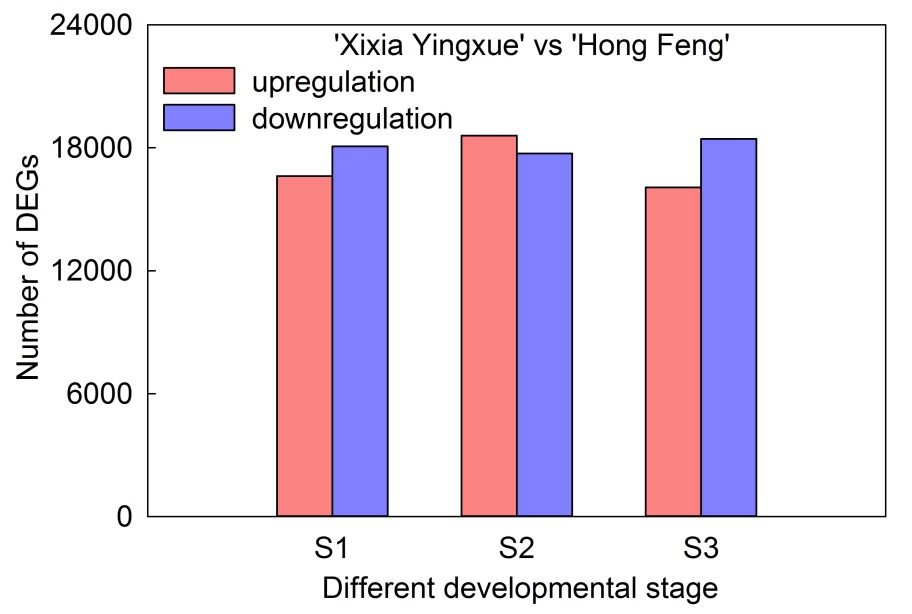


**Fig. S2.** Number of differentially expressed genes two *P. lactiflora* cultivar stems at three developmental stages. S1, flower-bud stage; S2, unfold-petal stage; S3, full-bloom stage.


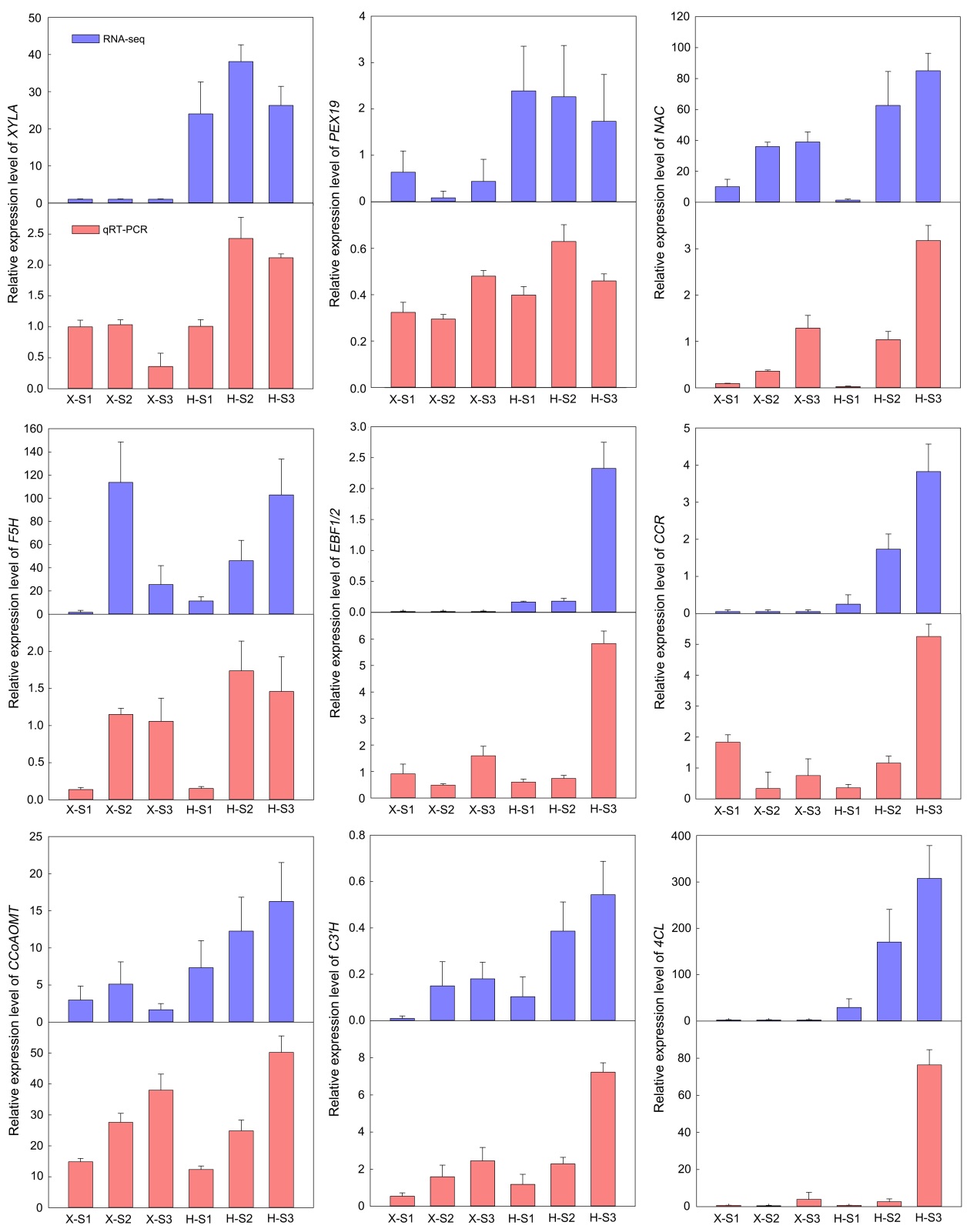


**Fig. S3.** Histogram of relative expression of nine selected genes based on RNA-seq and qRT-PCR data. X, Xixia Yingxue; H, Hong Feng; S1, flower-bud stage; S2, unfold-petal stage; S3, full-bloom stage. The values represent the means ± SDs.

**Table S1.** Primers used for qRT-PCR.

| Gene name | Forward primer (5'-3') | Reverse primer (5'-3') |
| --- | --- | --- |
| *Actin* | GTTGCCCTTGATTACGAG | CAGCTTCCATTCCGATTA |
| *C3'H* | CTGATAGGCATAGGAGTAGA | GAGAAAAGCTCAAGGGTA |
| *4CL* | TCACGCATCCAAACATCT | ATCTCATCCTCGGCAATC |
| *CCoAOMT* | GGGAGGTGACAGCCAAACAT | ATCATCGGGAAGAGCAAGG |
| *CCR* | GCAAAGAAGACTGAACACC | GTAACAGAAACAGGGGAT |
| *EBF1/2* | TCAATCAGCCGCAGCAAA | CAAGCCAAAATACTACCGA |
| *F5H* | AGCCTTCCAACCTCTATC | CTGGAGGGTATGGTAATC |
| *NAC* | AATAAGCCTCCTGGGTGT | TGTTGCCTTTGGGACTGT |
| *PEX19* | TTGATAAACTTCGGGAGC | CCATCATTCCATCCTTTC |
| *XYLA* | TTAGCACCAGGAGGATTC | TAGCAACATTTCGGAGTC |

**Table S2.** Statistics of the full-length transcriptome data.

| **Subreads** | **Number** |  |
| --- | --- | --- |
| Subreads number | 6,859,926 |  |
| Average subreads length (bp) | 1,766 |  |
| N50(bp) | 2,115 |  |
| **Classify** | |  |
| CCS | 624,682 |  |
| 5′-primer | 563,837 |  |
| 3′-primer | 571,603 |  |
| Poly-A | 529,846 |  |
| Full length | 478,428 |  |
| FLNC | 456,002 |  |
| Average FLNC read length (bp) | 1,987 |  |
| Consensus reads | 191,336 |  |
| **Correct** | **Before Correct** | **After Correct** |
| Total number | 191,336 | 191,336 |
| Mean length (bp) | 2,108 | 2,106 |
| Min length (bp) | 178 | 178 |
| Max length (bp) | 16,140 | 16,213 |
| N50 (bp) | 2,242 | 2,240 |
| N90 (bp) | 1,446 | 1,444 |
| **Cluster** | **Number of transcripts** | **Number of Genes** |
| <500 bp | 3,412 | 1,718 |
| 500–1 kbp | 9,958 | 6,164 |
| 1 k–2 kbp | 87,689 | 38,570 |
| 2 k–3 kbp | 65,515 | 46,506 |
| >3 kbp | 24,762 | 21,016 |
| Total | 191,336 | 113,974 |

**Table S3. Statistics of RNA-seq data.**

| Sample | Total clean reads  (M) | Total mapping  (%) | Uniquely mapping  (%) |
| --- | --- | --- | --- |
| Xixia Yingxue-S1-1 | 68.04 | 77.55 | 10.45 |
| Xixia Yingxue-S1-2 | 68.09 | 75.20 | 10.03 |
| Xixia Yingxue-S1-3 | 67.49 | 72.13 | 9.60 |
| Xixia Yingxue-S2-1 | 67.62 | 76.12 | 9.65 |
| Xixia Yingxue-S2-2 | 68.17 | 82.89 | 9.30 |
| Xixia Yingxue-S2-3 | 68.37 | 78.68 | 9.07 |
| Xixia Yingxue-S3-1 | 68.09 | 78.36 | 8.02 |
| Xixia Yingxue-S3-2 | 68.12 | 85.18 | 8.42 |
| Xixia Yingxue-S3-3 | 68.18 | 86.94 | 8.80 |
| Hong Feng-S1-1 | 68.28 | 76.46 | 12.18 |
| Hong Feng-S1-2 | 68.15 | 80.85 | 11.00 |
| Hong Feng-S1-3 | 68.19 | 77.63 | 11.81 |
| Hong Feng-S2-1 | 68.09 | 75.88 | 8.39 |
| Hong Feng-S2-2 | 67.12 | 84.06 | 9.64 |
| Hong Feng-S2-3 | 68.33 | 84.45 | 7.54 |
| Hong Feng-S3-1 | 67.17 | 84.22 | 8.34 |
| Hong Feng-S3-2 | 68.48 | 81.82 | 8.63 |
| Hong Feng-S3-3 | 68.35 | 86.02 | 8.83 |
| Average | 68.02 | 80.25 | 9.43 |

**Table S4.** Gene symbol of identified TFs

| Gene symbol | Sequence ID |
| --- | --- |
| *NAC1* | i2_HQ_PL_c126945/f2p0/2045 |
| *NAC3* | i1_LQ_PL_c40798/f1p5/1095 |
| *NAC5* | i1_LQ_PL_c81525/f1p1/1843 |
| *MYB1* | i1_LQ_PL_c41953/f1p0/1224 |
| *MYB2* | i1_LQ_PL_c194470/f1p1/1977 |
| *C3H1* | i2_HQ_PL_c130468/f2p31/2775 |
| *C3H2* | i1_HQ_PL_c105261/f2p6/1714 |
| *C3H3* | i3_LQ_PL_c12790/f1p0/3310 |
| *C3H4* | i2_LQ_PL_c14916/f1p0/2113 |
